# Comparison of Transcriptional Responses and Metabolic Alterations in Three Multidrug-Resistant Model Microorganisms, Staphylococcus aureus ATCC BAA-39, Escherichia coli ATCC BAA-196, and Acinetobacter baumannii ATCC BAA-1790, on Exposure to Iodine-Containing Nano-micelle Drug FS-1

**DOI:** 10.1128/mSystems.01293-20

**Published:** 2021-03-16

**Authors:** Ilya S. Korotetskiy, Sergey V. Shilov, Tatyana V. Kuznetsova, Aleksandr I. Ilin, Monique Joubert, Setshaba Taukobong, Oleg N. Reva

**Affiliations:** a Scientific Center for Anti-Infectious Drugs, Almaty, Kazakhstan; b Centre for Bioinformatics and Computational Biology, Department of Biochemistry, Genetics and Microbiology, University of Pretoria, Pretoria, South Africa; University of California, San Diego

**Keywords:** transcriptomics, *Escherichia coli*, *Staphylococcus aureus*, *Acinetobacter baumannii*, antibiotic resistance, iodine, nano-micelle

## Abstract

Iodine is one of the oldest antimicrobial agents. Until now, there have been no reports on acquiring resistance to iodine. Recent studies showed promising results on application of iodine-containing nano-micelles, FS-1, against antibiotic-resistant pathogens as a supplement to antibiotic therapy. The mechanisms of the action, however, remain unclear. The aim of this study was to perform a holistic analysis and comparison of gene regulation in three phylogenetically distant multidrug-resistant reference strains representing pathogens associated with nosocomial infections from the ATCC culture collection: Escherichia coli BAA-196, Staphylococcus aureus BAA-39, and Acinetobacter baumannii BAA-1790. These cultures were treated by a 5-min exposure to sublethal concentrations of the iodine-containing drug FS-1 applied in the late lagging phase and the middle of the logarithmic growth phase. Complete genome sequences of these strains were obtained in the previous studies. Gene regulation was studied by total RNA extraction and Ion Torrent sequencing followed by mapping the RNA reads against the reference genome sequences and statistical processing of read counts using the DESeq2 algorithm. It was found that the treatment of bacteria with FS-1 profoundly affected the expression of many genes involved in the central metabolic pathways; however, alterations of the gene expression profiles were species specific and depended on the growth phase. Disruption of respiratory electron transfer membrane complexes, increased penetrability of bacterial cell walls, and osmotic and oxidative stresses leading to DNA damage were the major factors influencing the treated bacteria.

**IMPORTANCE** Infections caused by antibiotic-resistant bacteria threaten public health worldwide. Combinatorial therapy in which antibiotics are administered together with supplementary drugs improving susceptibility of pathogens to the regular antibiotics is considered a promising way to overcome this problem. An induction of antibiotic resistance reversion by the iodine-containing nano-micelle drug FS-1 has been reported recently. This drug is currently under clinical trials in Kazakhstan against multidrug-resistant tuberculosis. The effects of released iodine on metabolic and regulatory processes in bacterial cells remain unexplored. The current work provides an insight into gene regulation in the antibiotic-resistant nosocomial reference strains treated with iodine-containing nanoparticles. This study sheds light on unexplored bioactivities of iodine and the mechanisms of its antibacterial effect when applied in sublethal concentrations. This knowledge will aid in the future design of new drugs against antibiotic-resistant infections.

## INTRODUCTION

Iodine was discovered by French chemist Bernard Courtois in 1811. Since then, it has been used as one of the most successful disinfectants, whose antimicrobial activity has never been compromised by any acquired or natural resistance. The history of its discovery and a comprehensive overview of the use of iodine in medicine were published in 1961 on the 150-year anniversary of the discovery of this chemical element (1). Iodine has, however, significantly lost its ground in medicine after the discovery of antibiotics by Alexander Fleming in 1928 (2, 3). Although they were originally developed for treating human infectious diseases, the remarkable antimicrobial properties of antibiotics have spread them to a broad application in farming, agroindustry, and aquaculture (4). The misuse and overuse of antibiotics have led to a strong selective pressure toward emerging and widespread distribution of antibiotic-resistant pathogens threatening public health systems worldwide (2, 4). Eventually, the situation with antibiotic resistance and problems with development of new antibiotics for an affordable price have come to a global crisis (5–8). An urgent need in alternative approaches to cope with the problem of antibiotic resistance is generally recognized (9, 10). It is of great importance to devise new strategies that focus on limiting, redirecting, and/or reversing the antibiotic resistance in bacteria.

Surprisingly, despite the use of iodine in medicine for over 200 years, the effect of iodine and its derivative compounds on metabolic processes and gene expression in living organisms remains basically unexplored except for numerous studies on iodine-rich thyroid hormones. A reason for this may be that iodine was used in medicine mostly for topical treatment of wounds or in composition of industrial disinfectants designed for a rapid eradication of germs, leaving no time for the treated bacteria to respond to the treatment by either gene regulation or metabolic adaptation. Only recently, several published works demonstrated rather complex mechanisms of the action of iodine on eukaryotic cells and gene expression patterns (11–13). An interesting fact was published by Klebanoff in 1967 (14) on the release of iodinated organic substances by leukocytes at spotted infections that suggested a possible natural therapeutic effect of iodine in concentrations much lower than that in the disinfectants. This discovery had no continuation in any further studies, probably because the iodine-based antimicrobials looked in the flourishing days of the antibiotic era as an unpromising archaic aftermath of 19th-century medicine. However, the postantibiotic era calls us to reconsider the applicability of previously abandoned anti-infection remedies (15).

Iodine-containing drugs seem to provide a viable option to overcome the antibiotic resistance problem, since they have strong antimicrobial properties and no acquired resistance to iodine has been reported (16–18). Molecular iodine is an effective mild and nontoxic Lewis acid catalyst (19) of great potential for synthesis of various macromolecular complexes (20) that can easily penetrate cells of microorganisms (21) and pass through bilipid layers of cell membranes (11). Since iodine can easily penetrate cell membranes, its application may be efficient against intracellular infections. This concept was implemented in a new drug, FS-1, developed as a new medicine against multidrug-resistant (MDR) tuberculosis (21, 22). FS-1 contains iodine molecules and ions bound to dextrin/oligopeptide nano-micelles. The general composition of FS-1 particles is shown below:

$$\left[\left\{{\left({\text{L}}_{n}{\left({\text{MeJ}}_{\text{3}}\right)}^{+}\right)}_{y}\right\}\{{[\text{Me}\left({\text{L}}_{m}\right)\text{J]}}^{+}{}_{x}\}{\left({\text{Cl}}^{-}\right)}_{y+x+k}\right]$$

where L is dextrin-polypeptide ligand; Me is Li/Mg ions; and *n*, *m*, *x*, *y*, and *k* are variable integers ≥1. Molecular mass of the micelles is in the range of 30 to 300 kDa.

FS-1 has passed both preclinical and clinical trials (www.clinicaltrials.gov, accession number NCT02607449) and was approved in Kazakhstan as a supplementary drug to be administered with the common antibiotics used against multidrug-resistant (MDR) and extensively drug-resistant (XDR) tuberculosis. It was demonstrated that the application of FS-1 increased the susceptibility of the treated bacteria to antibiotics (22); however, molecular mechanisms of this action remain unclear. As the use of multidrug-resistant Mycobacterium tuberculosis isolates for further studies was technically challenged, the study of the action of iodine-containing nano-micelles on antibiotic-resistant bacteria was continued using ATCC collection strains Escherichia coli BAA-196, Staphylococcus aureus BAA-39, and Acinetobacter baumannii BAA-1790. Complete genome sequences of these microorganisms were obtained using SMRT PacBio sequencing for two variants of the strains: the initial culture and the culture grown with subbactericidal concentrations of FS-1 in 10 daily passages (23–25). Global genetic variations, epigenetic modifications, and altered gene regulation in S. aureus BAA-39 and E. coli BAA-196 cultivated with FS-1 were analyzed in recent publications (26–28). The aim of the current study was to perform a systemic analysis of gene expression and metabolic alteration in these three selected multidrug-resistant pathogens affected by a 5-min exposure to the sublethal dose of the iodine-containing drug FS-1 in the late lagging phase and the middle of the logarithmic growth phase. Our expectation is that this study will shed light on unexplored bioactivities of iodine and that the comprehensive knowledge of metabolic and regulatory pathways affected by iodine-containing nano-micelles will aid in future design of new drugs against multidrug-resistant infections.

## RESULTS

The effect of a 5-min exposure to FS-1 on gene expression was analyzed on three model microorganisms: E. coli ATCC BAA-196, S. aureus ATCC BAA-39, and A. baumannii ATCC BAA-1790. Total RNA extracts were converted to cDNA and sequenced by the Ion Torrent technology. Then, the DNA reads were mapped against the complete genome sequences of these microorganisms. The drug FS-1 was applied at the end of the lagging growth phase (Lag experiment) and in the middle of the logarithmic growth phase (Log experiment). Growth curves estimated for the model microorganisms are shown in Fig. S1 in the supplemental material. Each experiment was repeated three times (with E. coli) or six times (with S. aureus and A. baumannii) to accumulate enough reads and repeats for achieving statistical confidence for the differentially expressed genes (Table 1). The counts of reads overlapping individual genes were normalized by the program Bioconductor DESeq2, and the statistical parameters of differential gene expression were calculated.

**TABLE 1:** Numbers of DNA reads after quality filtering and numbers of reads mapped against coding genes (CDS and ncRNA) and rRNA genes^*a*^

Strain	DNA reads	Exptl conditions			
		NC_lag	FS_log	NC_lag	FS_log
E. coli BAA-196 (three repeats)	Filtered reads	260,107	619,997	671,691	765,121
	Mapped to CDS and ncRNA	63,316 (24%)	110,878 (18%)	190,365 (28%)	207,353 (27%)
	Mapped to rRNA	163,496 (63%)	370,117 (60%)	253,911 (38%)	325,405 (43%)

S. aureus BAA-39 (six repeats)	Filtered reads	1,068,842	2,392,316	2,227,326	2,715,632
	Mapped to CDS and ncRNA	180,703 (17%)	538,815 (23%)	422,931 (19%)	591,975 (22%)
	Mapped to rRNA	615,263 (58%)	777,934 (33%)	1,301,357 (58%)	954,679 (35%)

A. baumannii BAA-1790 (six repeats)	Filtered reads	1,657,125	603,396	979,750	516,144
	Mapped to CDS and ncRNA	432,080 (26%)	94,377 (16%)	276,358 (28%)	163,271 (32%)
	Mapped to rRNA	574,373 (35%)	190,555 (32%)	186,081 (19%)	113,451 (22%)

a Negative-control (NC) and experimental (FS) samples obtained from the Lag and Log experiments are indicated in the column titles.

10.1128/mSystems.01293-20.1 FIG S1 Growth curves of E. coli ATCC BAA-196 (A), S. aureus ATCC BAA-39 (B), and A. baumannii ATCC BAA-1790 (C) calculated by the Baranyi and Roberts model implemented in the interactive DMFit application based on optical density (OD) values obtained in three repetitions using microarray plates with MH liquid medium. Average OD values are depicted by open circles. *R*-square values of deviations of predicted from recorded OD values are shown. Download FIG S1, PDF file, 1.0 MB. Copyright © 2021 Korotetskiy et al. 2021 Korotetskiy et al. https://creativecommons.org/licenses/by/4.0/ This content is distributed under the terms of the Creative Commons Attribution 4.0 International license.

Gene expression differences equal to or higher than 2-fold with estimated *P* values equal to or lower than 0.05 were considered significant and statistically reliable. Numbers of up- and downregulated genes in the Lag and Log growth phases in the three model microorganisms are shown in Table 2. Involvement of the differentially expressed genes in metabolic pathways was predicted by the program Pathway Tools v14.0. A summary of regulated metabolic pathways in the three model organisms is shown in Table 3. Also, all differentially expressed genes identified in E. coli, S. aureus, and A. baumannii are listed in Tables S1, S2, and S3, respectively. Involvements of these genes in metabolic pathways predicted by the program Pathway Tools are indicated, also.

**TABLE 2:** Numbers of regulated genes identified in the three model microorganisms treated with FS-1 in the Lag and Log growth phases

Strain	Lag growth phase		Log growth phase	
	Upregulated	Downregulated	Upregulated	Downregulated
E. coli ATCC BAA-196	42	78	15	43
S. aureus ATCC BAA-39	223	230	76	29
A. baumannii ATCC BAA-1790	102	79	78	127

**TABLE 3:** Regulation of metabolic pathways by the treatment with FS-1 of E. coli ATCC BAA-196, S. aureus ATCC BAA-39, and A. baumannii ATCC BAA-1790 at the Lag and Log growth phases^*a*^

Pathway^*b*^	BAA-196		BAA-39		BAA-1790	
	Lag	Log	Lag	Log	Lag	Log
Central metabolism						
Glycolysis/gluconeogenesis	−1	1	−1	1	-	1
Entner-Doudoroff pathway	-	1	-	-	-	-
TCA (prokaryotic)	−1	−1	−1	-	1	1
Glyoxylate cycle	−1	−1	-	-	1	1
Pyruvate decarboxylation to acetyl-CoA	−1	-	1	-	-	−1

Respiration and electron transfer						
Formate to dimethyl sulfoxide electron transfer	-	1	-	-	-	-
NADH to cytochrome *bd* oxidase electron transfer	-	-	1	-	0	−1
NADH to cytochrome *bo* oxidase electron transfer	-	-	-	-	1	-
Proline to cytochrome *bo* oxidase electron transfer	-	-	-	-	−1	−1
Respiration (aerobic, cytochrome *c*)	−1	-	-	-	1	-
Respiration (anaerobic)	−1	−1	−1	-	1	−1
Succinate to cytochrome *bd* oxidase electron transfer	−1	-	1	-	−1	−1
Succinate to cytochrome *bo* oxidase electron transfer	−1	-	-	-	-	-

Fermentation						
Acetyl-cofermentation to butyrate II	−1	−1	1	-	0	0
Conversion of succinate to propionate	-	-	-	-	-	−1
Crotonate fermentation (to acetate and cyclohexane carboxylate)	-	-	−1	-	-	-
Heterolactic fermentation	-	-	0	1	-	-
Lysine fermentation to acetate and butyrate	-	-	-	-	0	0
Mixed acid fermentation	−1	−1	0	-	1	-
Nitrate reduction (dissimilatory)	−1	-	1	-	-	-
Pyruvate fermentation to acetate	-	-	1	-	-	−1
Pyruvate fermentation to lactate	-	-	-	-	-	1

Amino acid biosynthesis						
Alanine biosynthesis	-	−1	-	-	-	-
Arginine biosynthesis	-	-	−1	−1	-	−1
Glutamate biosynthesis	-	-	−1	−1	-	1
Glutamine biosynthesis	-	-	1	-	-	-
Homoserine and methionine biosynthesis	-	-	−1	-	1	1
Leucine, valine, and isoleucine biosynthesis	−1	-	−1	-	1	1
Lysine, threonine, and methionine biosynthesis I	-	-	−1	-	0	1
Ornithine biosynthesis	-	-	−1	−1	-	−1
Proline biosynthesis	-	-	-	-	0	-

Amino acid degradation						
Alanine degradation II (to d-lactate) and IV	-	-	−1	-	-	−1
Arginine degradation pathways II, III, and V	1	-	−1	-	−1	−1
Asparagine degradation	-	-	-	-	-	−1
Glutamate degradation	−1	-	−1	-	-	−1
Glutamine degradation	-	-	0	−1	-	−1
Histidine degradation	-	-	−1	-	−1	−1
Isoleucine degradation	-	-	-	-	1	1
Lysine degradation	-	-	−1	-	-	-
Methionine degradation I (to homocysteine)	-	-	-	-	-	−1
Proline degradation	-	-	−1	-	−1	−1
Threonine degradation	-	-	−1	-	-	-
Tyrosine degradation	-	-	-	-	−1	−1
Valine degradation	-	-	1	-	1	1

Nucleotide biosynthesis						
Adenosine and guanosine deoxyribonucleotide *de novo* biosynthesis	1	-	1	-	-	-
Adenosine ribonucleotide *de novo* biosynthesis	-	-	1	-	-	-
CMP phosphorylation	-	-	1	-	-	-
Pyrimidine deoxyribonucleotide phosphorylation	-	-	1	-	-	-
Pyrimidine deoxyribonucleotide *de novo* biosynthesis	1	1	0	−1	−1	1
Thiamine diphosphate biosynthesis III (*Staphylococcus*)	-	-	-	1	-	-
UMP biosynthesis	-	-	−1	−1	-	-
UTP and CTP *de novo* biosynthesis	-	-	1	-	−1	-

Nucleotide salvage						
Guanine and guanosine salvage	-	-	-	-	1	-
Pyrimidine deoxyribonucleoside salvage	-	1	1	-	-	-
Pyrimidine nucleobase salvage	-	-	1	-	−1	-
Thiamine salvage	-	-	-	1	-	-
Xanthine and xanthosine salvage	-	-	-	-	1	-

Nucleotide degradation						
Purine deoxyribonucleoside degradation	−1	-	−1	-	-	-
Purine nucleobase degradation II (anaerobic)	-	-	0	-	-	-
Pyrimidine deoxyribonucleoside degradation	−1	-	−1	-	-	-
Pyrimidine ribonucleoside degradation	−1	-	-	-	-	-
Uracil degradation	−1	-	-	-	-	-

Fatty acid and lipid biosynthesis						
(KDO)2-lipid biosynthesis	1	-	-	-	-	-
Acetate conversion to acetyl-CoA	-	−1	−1	-	-	-
Acyl carrier protein activation	-	-	−1	-	-	-
CDP-diacylglycerol (phospholipid) biosynthesis	-	-	-	-	-	−1
Fatty acid activation	-	−1	−1	-	−1	-
Fatty acid biosynthesis initiation and lipid biosynthesis	1	-	-	-	−1	−1
Fatty acid elongation/saturated	-	-	1	-	−1	-
Palmitate biosynthesis	-	-	1	-	-	-
Phosphatidylglycerol and phospholipid biosynthesis	-	-	-	-	-	−1
Stearate biosynthesis	-	-	1	-	−1	-

Fatty acid oxidation and degradation						
Fatty acid beta-oxidation (aerobic)	−1	−1	−1	-	1	1
Fatty acid beta-oxidation (anaerobic)	−1	−1	−1	-	−1	−1
Octane oxidation	-	-	-	-	−1	−1
Oleate beta-oxidation	-	-	-	-	1	-
2-Oxoglutarate decarboxylation to succinyl-CoA	−1	-	1	-	-	-
2-Oxoisovalerate decarboxylation to isobutanoyl-CoA	-	-	1	-	-	-
Acetate formation from acetyl-CoA	-	-	1	-	-	-
Acetoin degradation	-	-	-	-	-	1
Androstenedione degradation (steroid metabolism)	−1	-	-	-	-	-
Ethanol degradation	−1	−1	0	-	−1	−1
Glycerol and glycerophosphodiester degradation	−1	-	1	-	-	-
Glycerol degradation to butanol	-	1	-	-	-	-
Glycol metabolism and degradation	−1	-	-	-	-	-
Oxalate degradation	1	-	-	-	-	-
Triacylglycerol degradation	-	-	−1	-	-	-

Cell wall and cytoplasmic membrane						
Anhydromuropeptide recycling	-	1	0	−1	-	-
*cis*-Dodecenoyl biosynthesis	-	-	1	-	-	-
*cis*-Vaccenate biosynthesis	-	-	1	-	-	-
Di-*trans*, poly-*cis*-undecaprenyl phosphate biosynthesis	-	-	-	-	-	−1
GDP-mannose (glycoprotein) biosynthesis	-	-	-	-	-	1
Lipopolysaccharide biosynthesis	1	-	-	-	-	-
Muropeptide degradation	-	1	-	-	-	-
*N*-Acetylglucosamine, *N*-acetylmannosamine, and *N*-acetylneuraminate degradation	-	-	-	−1	-	-
Peptidoglycan biosynthesis	-	-	0	-	1	1
Teichoic acid (poly-glycerol) biosynthesis	-	-	−1	-	-	-
Cofactor and vitamin biosynthesis						
[2Fe-2S] iron-sulfur cluster biosynthesis	-	−1	-	-	-	-
1,4-Dihydroxy-2-naphthoate (quinol and quinone) biosynthesis	-	-	1	-	-	-
8-Amino-7-oxononanoate (biotin) biosynthesis	-	-	1	-	−1	-
Biotin biosynthesis	-	-	1	-	−1	-
Biotin-carboxyl carrier protein assembly	1	-	1	-	−1	-
Flavin biosynthesis	-	-	-	-	1	−1
Folate transformations	-	-	−1	−1	-	-
Formate reduction to 5,10-methylenetetrahydrofolate	-	-	−1	-	-	-
Formyl-THF biosynthesis	-	1	0	−1	-	-
Glycine betaine biosynthesis	-	-	-	1	−1	-
l-Ascorbate biosynthesis III	-	-	-	-	-	-
Menaquinol-7 biosynthesis	-	-	1	-	-	1
Molybdenum cofactor biosynthesis	-	−1	1	1	-	-
NAD salvage pathway I	-	-	1	-	-	−1
Pantothenate biosynthesis	-	-	-	1	-	-
Precursor of queuosine-tRNA (PreQ0) biosynthesis	-	-	1	1	-	-
*S*-Adenosyl-l-methionine biosynthesis	-	-	1	-	1	1
Tetrahydrobiopterin salvage	-	-	-	-	-	0
Tetrahydrofolate biosynthesis	-	-	-	1	-	1
Tetrahydrofolate salvage from 5,10-methenyltetrahydrofolate	-	-	−1	-	-	-
Thiamine diphosphate biosynthesis	-	−1	-	-	-	-
Thiazole biosynthesis	-	−1	-	-	-	-

Utilization of organic and inorganic compound						
Acetate utilization and formation	-	−1	0	-	-	-
Ammonia assimilation cycle	-	-	0	−1	-	−1
CO_2_ fixation into oxaloacetate (anapleurotic)	−1	−1	-	-	-	-
Formaldehyde assimilation I (serine pathway)	-	-	−1	1	-	-
Nitrate reduction (assimilatory)	−1	-	-	-	-	−1
Selenate reduction	-	-	-	-	-	−1
Sulfate utilization and metabolism	-	-	-	-	-	−1
Two-component alkanesulfonate monooxygenase (sulfur metabolism)	-	-	-	-	-	−1
Urea degradation	-	-	1	-	-	-

Protein synthesis and modification						
Protein citrullination	-	-	−1	-	-	-
tRNA charging	1	1	1	-	-	-
tRNA processing	1	-	-	-	-	-

Synthesis of carbohydrates						
2-Keto-l-gulonate (carbohydrate) biosynthesis	-	-	-	-	-	1
ADP-l-glycero-beta-d-manno-heptose (carbohydrate) biosynthesis	1	-	-	-	-	-
UDP-d-galactose biosynthesis	-	-	-	-	-	1
UDP-*N*-acetyl-alpha-d-fucosamine biosynthesis	-	-	-	−1	-	-
UDP-*N*-acetyl-alpha-d-quinovosamine biosynthesis	-	-	-	−1	-	-
UDP-*N*-acetyl-d-glucosamine biosynthesis I	-	-	−1	-	-	-
UDP-*N*-acetylmuramoyl-pentapeptide biosynthesis I (meso-DAP-containing)	-	-	0	-	1	-
UDP-*N*-acetylmuramoyl-pentapeptide biosynthesis II (lysine-containing)	-	-	0	-	-	-

Synthesis of secondary metabolites						
2,3-Dihydroxybenzoate (aromatic compound) biosynthesis	-	-	−1	-	-	-
4,4′-Diapolycopenedioate (terpenoid/carotenoid) biosynthesis	-	-	−1	−1	-	-
Ethylene biosynthesis	-	-	-	-	-	1
Heme biosynthesis from glutamate	1	-	1	-	−1	-
Indole-3-acetate (auxin) biosynthesis	-	-	-	-	-	1
Methylerythritol phosphate and mevalonate pathways (isoprenoid biosynthesis)	-	-	-	−1	−1	-
Myoinositol biosynthesis	1	-	−1	−1	-	-
Putrescine biosynthesis	1	-	-	-	-	-
Degradation of carbohydrates						
l-Lactaldehyde degradation (aerobic)	-	-	−1	-	−1	0
2′-Deoxy-alpha-d-ribose 1-phosphate degradation	−1	-	−1	-	-	-
Chitin degradation	-	-	−1	-	-	-
d-Galactosamine and *N*-acetyl-d-galactosamine degradation	−1	-	-	-	-	-
d-Gluconate degradation	-	-	−1	-	−1	-
Fructose degradation	−1	-	1	-	-	-
l-Rhamnose degradation	−1	-	-	-	-	-
Ribose degradation	−1	-	-	−1	-	-
Trehalose degradation (low osmolarity)	-	-	−1	-	-	-

Degradation of other compounds						
2-Methylcitrate cycle (propanoate degradation)	-	−1	-	-	−1	-
Acrylonitrile degradation	-	-	-	-	-	1
Amine and polyamine degradation	−1	-	−1	-	−1	−1
Aromatic compound degradation via beta-ketoadipate	-	-	-	-	1	−1
Cyanate degradation	−1	−1	-	-	-	-
Ethylene glycol degradation	−1	-	-	-	-	-
Gallate degradation (anaerobic)	-	-	1	-	-	-
Ketolysis	-	-	-	-	1	1
Methanesulfonate degradation	-	-	-	-	-	−1
Methylglyoxal degradation	-	-	1	-	−1	−1
Phenylacetate degradation (aerobic)	−1	-	-	-	1	-
Phenylethylamine degradation	−1	-	-	-	−1	−1
Phenylmercury acetate degradation	-	-	1	-	-	-
Protocatechuate degradation (ortho-cleavage pathway)	-	-	-	-	1	−1
Taurine degradation	-	-	-	-	-	1

Stress response and resistance						
Arsenate detoxification by glutaredoxin	-	-	-	-	1	-
ppGpp biosynthesis	1	-	−1	-	-	-
Superoxide radical degradation	-	-	1	-	-	-
Thioredoxin pathway	-	-	−1	-	-	-

a Regulation was predicted by the program Pathway Tools v14.0 for the regulated genes (see Tables S1 to S3). Upregulation is depicted by 1; downregulation is depicted by −1; alternative regulation of genes involved in the pathway is depicted by 0.

b Abbreviations: KDO, 2-keto-3-deoxyoctulosonic acid; THF, tetrahydrofolate; DAP, diaminopimelic acid.

10.1128/mSystems.01293-20.3 TABLE S1 Genes regulated in E. coli ATCC BAA-196 in the Lag and Log growth phases by 5-min exposure to FS-1. Download Table S1, XLSX file, 0.04 MB. Copyright © 2021 Korotetskiy et al. 2021 Korotetskiy et al. https://creativecommons.org/licenses/by/4.0/ This content is distributed under the terms of the Creative Commons Attribution 4.0 International license.

10.1128/mSystems.01293-20.4 TABLE S2 Genes regulated in S. aureus ATCC BAA-39 in the Lag and Log growth phases by 5-min exposure to FS-1. Download Table S2, XLSX file, 0.08 MB. Copyright © 2021 Korotetskiy et al. 2021 Korotetskiy et al. https://creativecommons.org/licenses/by/4.0/ This content is distributed under the terms of the Creative Commons Attribution 4.0 International license.

10.1128/mSystems.01293-20.5 TABLE S3 Genes regulated in A. baumannii ATCC BAA-1790 in the Lag and Log growth phases by 5-min exposure to FS-1. Download Table S3, XLSX file, 0.06 MB. Copyright © 2021 Korotetskiy et al. 2021 Korotetskiy et al. https://creativecommons.org/licenses/by/4.0/ This content is distributed under the terms of the Creative Commons Attribution 4.0 International license.

**FIG 1:**
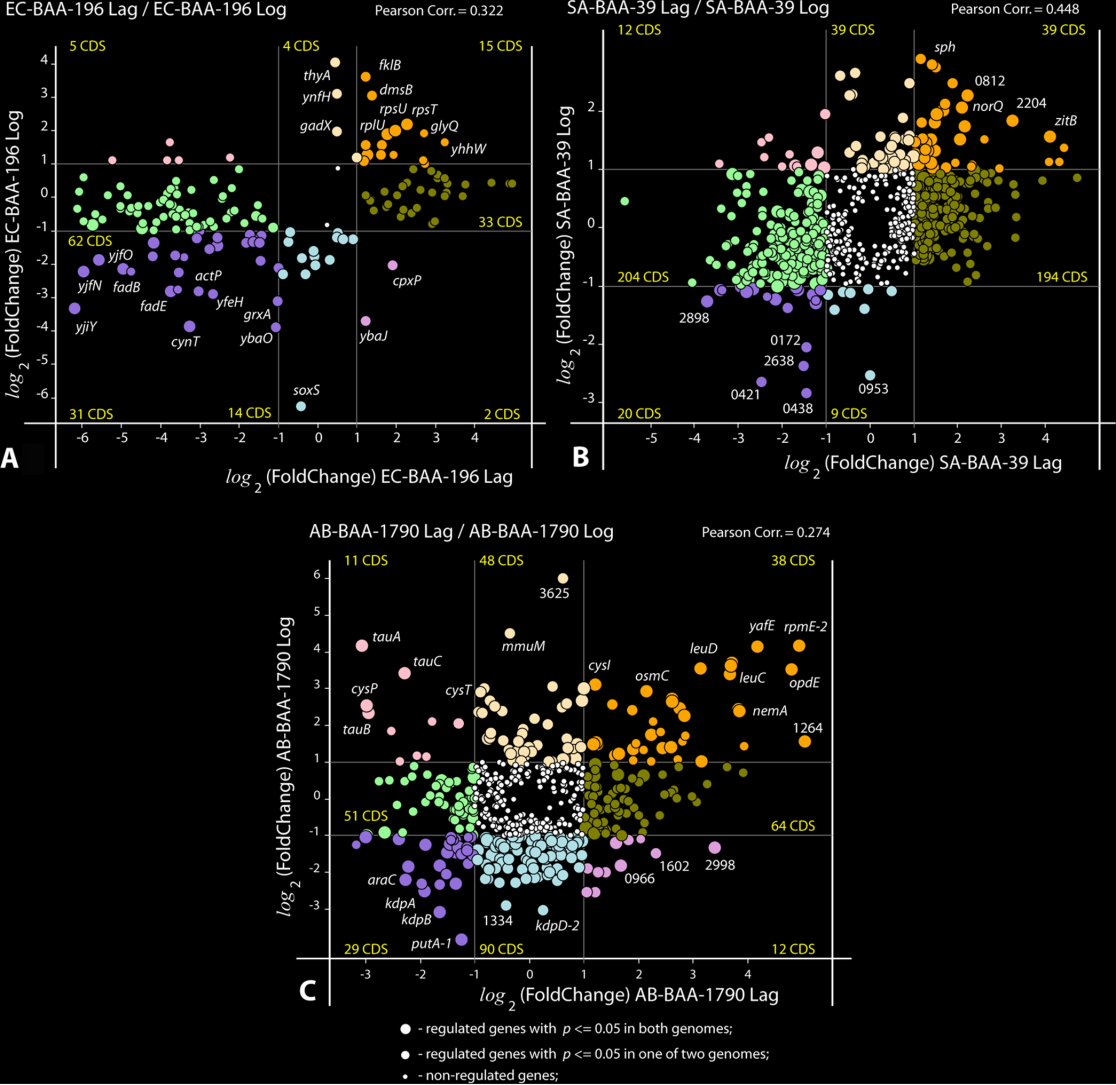
Plots of coregulation of genes in the Lag and Log growth phases. (A) E. coli BAA-196 (EC-BAA-196 Lag/Log); (B) S. aureus BAA-39 (SA-BAA-39 Lag/Log); (C) A. baumannii BAA-1790 (AB-BAA-1790 Lag/Log). Circles represent protein coding genes (CDS) plotted according to their negative and positive log_2_(fold change) values calculated in the Lag experiment (*x* axis) and Log experiment (*y* axis). The outermost regulated genes are labeled by their names or locus tag numbers. Thin vertical and horizontal lines within the plots separate genes with 2-fold or higher regulation and split the plots into sectors of genes of different categories depending on their coregulation. Numbers of CDS falling into different sectors are shown. Up- and down-coregulated genes, oppositely regulated genes, and the genes regulated only in one experiment are depicted by different colors. Statistical reliability of the fold change predictions is depicted by sizes of circles as explained in the legend at the bottom of the figure. Estimated Pearson correlation coefficients are given on the top of each plot.

The gene regulation caused by the treatment with FS-1 at different growth phases showed some level of coregulation with aggregated Pearson correlation coefficients of 0.504, 0.441, and 0.259 calculated under the Lag and Log experimental conditions for all differentially expressed genes identified for the strains of E. coli, S. aureus, and A. baumannii, respectively (Fig. 1). In the case with A. baumannii, the correlation was smaller due to an opposite regulation of several genes involved in taurine, sulfate, and nitrate transportation (*tauAB*, *cysA*, *cysP*, *cysW*, *ybaN*), which were significantly downregulated by the treatment with FS-1 in the Lag growth phase but upregulated in the Log phase under the same condition. In S. aureus, an alternative regulation was observed for gene *pyrC* involved in pyrimidine *de novo* biosynthesis.

### Differential gene expression in E. coli ATCC BAA-196.

**FIG 2:**
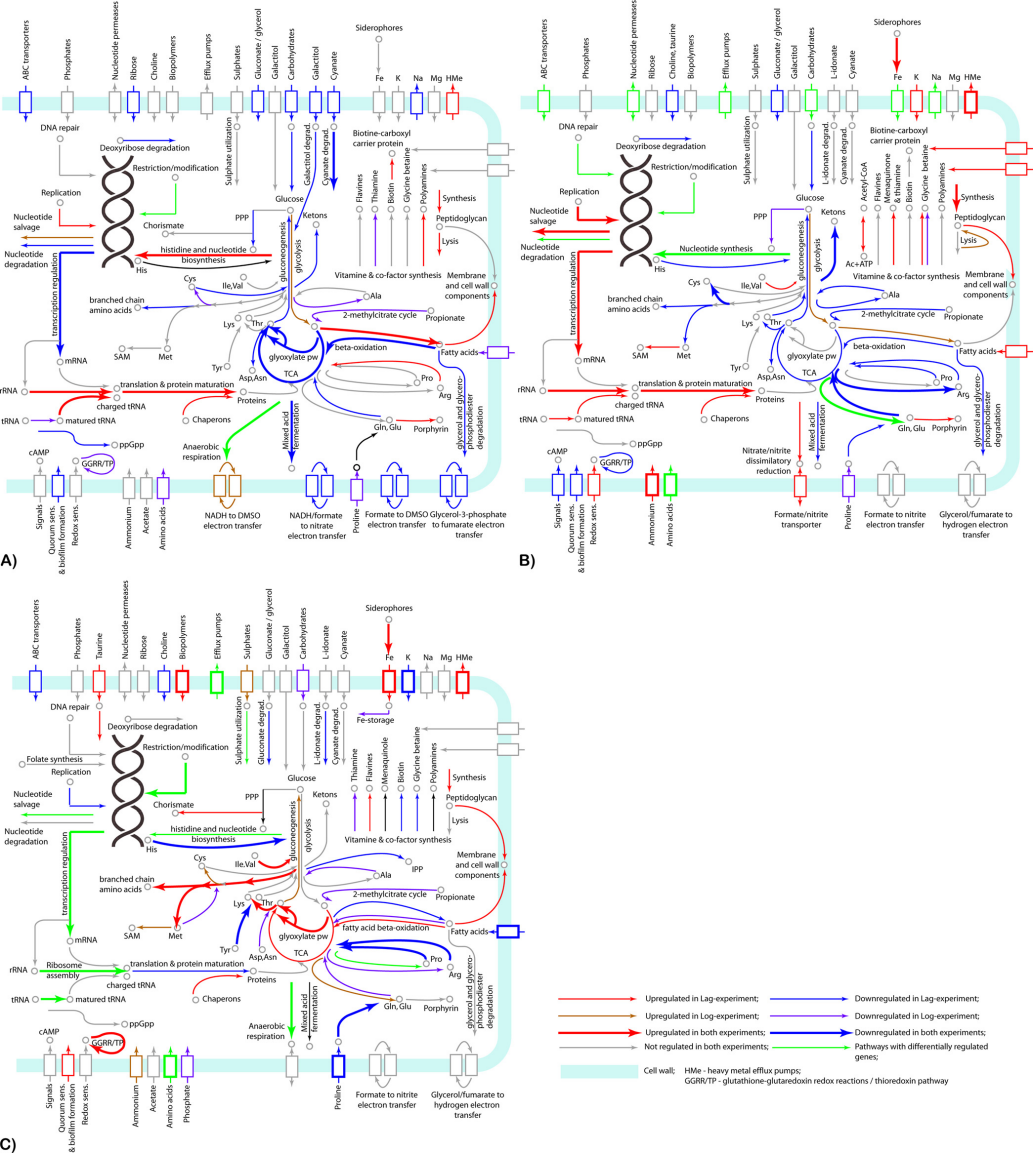
Metabolic pathways affected by the treatment of the model microorganisms E. coli BAA-196 (A), S. aureus BAA-39 (B), and A. baumannii BAA-1790 (C) with FS-1 as predicted by gene expression patterning in the Lag and Log experiments. Up- and downregulation of metabolic pathways discovered in both experiments, or only in one experiment, are depicted by arrows of different colors and widths as explained in the legend at the bottom right of the figure. Cell membrane and cell wall-associated proteins are shown by blocks depicted by the same color scheme. Individual pathways and key compounds are labeled.

Differential gene expression in E. coli treated for 5 min with FS-1 in the Lag and Log growth phases is shown in Table S1. The scheme of regulated metabolic pathways is shown in Fig. 2A.

The treatment with FS-1 affected the central metabolism of the bacterium. The genes of the tricarboxylic acid (TCA) cycle, *fumA*, *sdhA*, *sdhB*, *sucA*, *sucB*, and *sucC*, were strongly downregulated at least in one growth phase. The glyoxylate shunt genes *acnB* and *gltA* were downregulated in the Log phase. Glycolytic gene *pfkA* was 3-fold upregulated in the Lag and Log experiments, and another glycolytic gene, *tpiA*, was 3-fold upregulated in the Log phase. All these observations indicate a general trend of an activation of glycolysis in the FS-1-treated E. coli. Strong suppression of the TCA cycle with glycolysis activation is indicative for anaerobiosis (29). A complete switch to anaerobiosis was reported for this strain cultivated for 10 passages with FS-1 as reported in the previous study (27, 28). In this study, a strong upregulation of NADH:quinone oxidoreductase *ndh* was observed in both growth phases. This enzyme is important for aerobic and anaerobic respiration. It participates in transportation of protons from NADH to oxygen through a cascade of H^+^-transporting ubiquinol oxidases CyoABCD (these genes were insignificantly downregulated by FS-1) or CydABXH (insignificantly upregulated), or to nitrates through ubiquinol:nitrate oxidoreductases NarGHI (*narG* was 6.6-fold downregulated, *P* = 0.09). Mixed acid fermentation enzymes (*acnB*, *adhE*, *gltA*, *fdhF*, *frdA*, *frdB*) and all other electron transfer systems were mostly downregulated, namely, formate-to-dimethyl sulfoxide (DMSO)/nitrite/trimethylamine *N*-oxide (*fdoGH*), glycerol-3-phosphate (G3P)-to-cytochrome *bo* oxidase (*glpD*), G3P-to-fumarate/hydrogen peroxide reductases (*glpAB*, *frdA*), NADH/hydrogen-to-fumarate reductase (*frdA*), and succinate-to-cytochrome *bd* oxidase (*sdhAB*). A sole exception was the upregulated NADH-to-DMSO electron transfer system catalyzed by a complex of dimethyl sulfoxide reductase, DmsABC, with menaquinol. In the Log phase, *dmsB* was 8-fold upregulated. It suggests a possible transition of the FS-1-treated E. coli toward anaerobic respiration using DMSO as a terminal electron acceptor.

Many catabolic processes and transmembrane transportation systems were inhibited in the FS-1-treated cells. Downregulation was observed for the genes involved in carbohydrate transportation (*lamB*, *malE*, *malK*, *mglABC*, *treB*) and degradation (*fruK*), acetate/glycolate permease *actP*, galactitol transportation and degradation (*gatYZ*), cyanate transportation and degradation (*cynS*, *cynT*, *cynX*), glycerol and glycero-phosphodiester degradation (*glpABDKO*, *dhaKL*), propanoate degradation through the 2-methylcitrate cycle (*acnB*, *acs*, *fadD*), pyrimidine and deoxyribose degradation (*deoAC*), fatty acid transportation (*fadL*), and fatty acid beta-oxidation by the aerobic (*fadBDE*) and anaerobic (*fadIJ*) pathways. Several anabolic pathways were activated including fatty acid, lipid, and lipopolysaccharide biosynthesis (*accD*, *lpxA*, *rfaD*), peptidoglycan synthesis and recycling (*glmS*, *spr*), synthesis of polyamines (*speB*), nucleotide and histidine biosynthesis (*guaB*, *nrdA*, *thyA*), and porphyrin synthesis from glutamate (*hemL*). Synthesis and degradation of amino acids remained either unaffected or inhibited. Genes encoding multiple ribosomal proteins (*rimM*, *rplI*, *rplL*, *rplU*, *rpsT*, *rpsU*, *rpmA*, *yfgB*) and the protein translation/maturation genes (*efp*, *fklB*) were significantly upregulated, which may indicate an increased protein synthesis. The activated anabolism combined with the inhibition of the nutrient uptake systems could lead to a general starvation and catabolic repression by the (p)ppGpp alarmone system that is activated by an increased concentration of uncharged tRNA (30, 31). To prevent the stringent response, the genes encoding multiple tRNA charging enzymes (*argS*, *glnS*, *glyQ*, *glyS*, *tyrS*) and *spoT* ppGpp hydrolase were upregulated by the treatment with FS-1.

**FIG 3:**
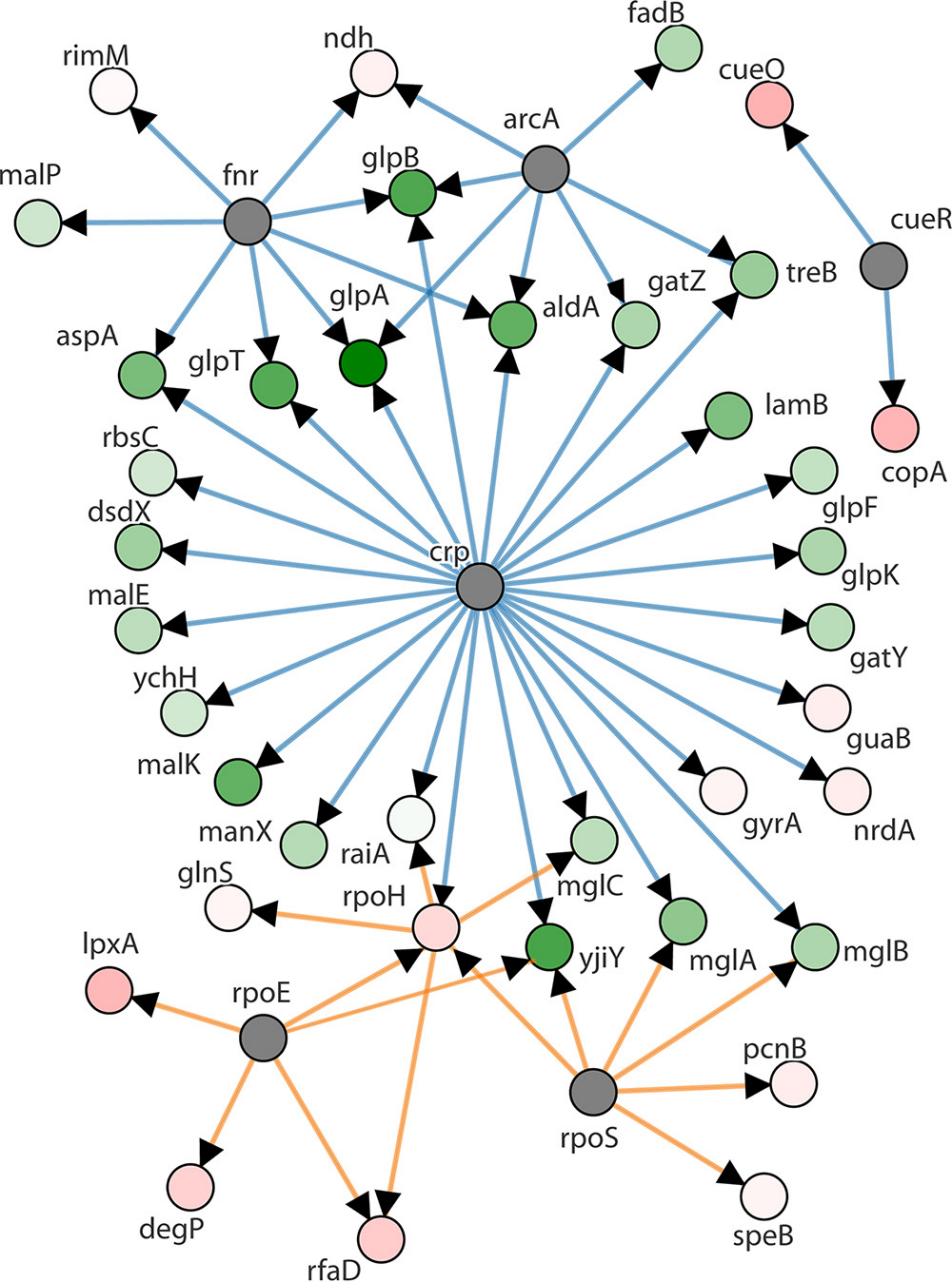
PheNetic network of the regulated genes of E. coli BAA-196 identified in the Lag experiment and clustered according to their regulation by higher-level transcriptional regulators. Upregulated genes are depicted by pink nodes, and downregulated genes are indicated by green nodes (vertices). Color intensity indicates levels of the expression fold changes. Gray nodes are transcriptional regulators involved in the network, whose expression was not reliably changed. Orange edges show regulation by transcriptional activators, and blue edges show regulation by transcriptional repressors. Direct regulation by the transcriptional regulators is indicated by arrowheads.

The regulatory network of differential gene expression in the FS-1-treated E. coli was modeled by the program PheNetic (Fig. 3). Differentially expressed genes of E. coli belong to several regulons controlled by DNA-binding transcriptional dual regulators cAMP receptor protein (CRP), fumarate and nitrate reduction (FNR) regulatory protein, and ArcA, which are involved in transition from aerobic to anaerobic growth; by three interdependent stress response sigma factors, RpoE, RpoS, and RpoH; and by the osmotic/heavy metal stress response DNA-binding transcriptional regulator CueR. The latter regulator controls the expression of copper transporter CopA and multicopper oxidase CueO, which were significantly upregulated in the FS-1-treated E. coli. CRP regulator acts mostly as a transcriptional repressor activated by cAMP in response to the osmotic stress (32). Also, it was reported that cAMP-CRP complex regulates the central metabolism and carbon source utilization under unavailability of glucose (33).

### Differential gene expression in S. aureus ATCC BAA-39.

Gene regulation in S. aureus treated with FS-1 in the Lag and Log growth phases is shown in Table S2. The scheme of the regulated metabolic pathways is shown in Fig. 2B.

The treatment with FS-1 suppressed in S. aureus the TCA pathway genes (*icd*, *sucC*) and activated glycolysis (*gpmI*, *pgk*, *tpiA*). This may indicate a possible transition to anaerobiosis (29); however, the *cydA* gene encoding cytochrome *bd*-I ubiquinol oxidase involved in the oxidative phosphorylation process was significantly activated in the FS-1-treated S. aureus. Cytochrome *bd*-I terminal oxidase catalyzes the reduction of NADH molecules by molecular oxygen or oxygen-rich compounds such as nitrates and nitrites. Genes *nirB* and *narGH* encoding nitrite/nitrate reductases involved in dissimilatory nitrite reduction for anaerobic respiration were significantly upregulated in the FS-1-treated S. aureus in the Lag growth phase. Another upregulated gene related to anaerobiosis is *fnt* encoding bidirectional formate-nitrite transporter. Fnt plays an important role in regulation of levels of intracellular formate during anaerobic respiration and in providing intracellular dissimilatory nitrite reductases with nitrite. Activation of this transporter may be a preparatory step for anaerobiosis; however, the key enzyme of the anaerobic metabolism, pyruvate:formate lyase PflB, which coverts pyruvate to acetyl coenzyme A (acetyl-CoA) and formate, was downregulated in the treated S. aureus. Acetyl-CoA synthesis from acetate was activated by an upregulation of acetate kinase *ackA*. However, this enzyme may work in the opposite direction, catalyzing ATP production from acetyl-CoA. One of the major sources of acetyl-CoA in cells is beta-oxidation of fatty acids. This pathway was inhibited in the FS-1-treated S. aureus (Lag-phase experiment) by downregulation of genes *fadA* (2.5-fold) and *fadB* (7.5-fold). In contrast, fatty acid biosynthetic enzyme acetyl-CoA carboxyltransferase *accD* was 1.7-fold upregulated. All these genes were not regulated in the Log-phase experiment. The analysis of the gene expression pattern suggests that *ackA* was activated for acetyl-CoA production. Also, this gene plays an important role for S. aureus in maintenance of metabolic homeostasis under stressful conditions (34).

Amino acid biosynthesis was generally downregulated in the FS-1-treated culture except for the glutamate biosynthesis via glutamine (*glnA*, *gltD*) followed by porphyrin biosynthesis (*hemC*) that in S. aureus is associated with growth under anaerobic conditions (35). Activated synthesis of menaquinone by upregulation of genes *menD* and *menH* also may be associated with anaerobiosis as the encoded compounds are parts of the NADH:menaquinone electron transfer system controlled by NADH oxidoreductase (36).

*De novo* purine (*purA*) and pyrimidine (*pyrG*, *thyA*) biosynthesis and salvage (*guaC*) genes were activated in the Lag-phase experiment, as well as the xanthine permease *pbuX* and the hypoxanthine/guanine permease *pbuG*, while the uracil:proton symporter *pyrP* was strongly downregulated. Gene *deoB* associated with nucleotide degradation was downregulated. DNA repair proteins were differentially regulated. Methyl-directed mismatch repair complex *mutSL*, modified pyrimidine reparative endonuclease *nth*, UV DNA repair and Holliday junction renaturation helicases *pcrA* and *ruvB*, RNA decay-controlling helicase *cshB*, negative supercoil relaxase *topA*, superoxide dismutase *sodA*, and DNA recombination gene *radA* were strongly upregulated. In contrast, SOS-response genes *recA*, *lexA*, and *uvrAB*, responsible for repair of double-strand DNA breaches, were downregulated.

Many genes controlling the accuracy of protein synthesis were activated in the FS-1-treated S. aureus. These genes involve tRNA charging (*aspS*, *gltX*, *hisS*, *ileS*, *leuS*, *pheS*, *valS*) and tRNA processing (*gatA*, *gatC*, *miaB*), genes for ribosomal proteins (*rimM*, *rlmB*, *rplJ*, *rpmG*, *rpmH*, *rpsP*, *yjbO*), RNA transcription terminator *rho*, protein release factor *prfC*, protein translocase *secG*, and transpeptidase-transglycosylase *sgtA*. In contrast, the ribosomal inhibitor *raiA*, ribosomal methyltransferases *mraW* and *rlmH*, and protein uptake translocase *secA2* were downregulated. Multiple chaperon coding genes, *copZ*, *dnaJ*, *dnaK*, *groL*, and *hslO*, were significantly upregulated.

In contrast to E. coli, transmembrane transportation proteins generally were not suppressed by the treatment with FS-1 or differentially regulated. The branched-chain amino acid transporter *brnQ*, amino acid permease *lysP2*, cysteine transporter *tcyAB,* and histidine transporter *yuiF* were upregulated, whereas d-serine/d-alanine/glycine transporter *cycA1*, glutamine transporter *glnQ*, and amino acid permease *gabP* were downregulated. Carbohydrate, organic acid, and fatty acid transporters *fruA* (fructose), *scrA* (sucrose), *lctP2* (l-lactate), and *msbA* (lipids) were activated, whereas mannitol transporter *mtlA*, oligopeptide transporters *oppA2*, *oppD*, and *oppF3*, trehalose transporter *treP*, and choline transporters *opuBA* and *opuBB* were inhibited. The multidrug resistance efflux pump *norA*, multiple heavy metal efflux pumps (*copA*, *czrA*, *mntH*, *nikE*, *zitB*), putative sugar efflux transporter HMPRNC0000_0740, and auxin efflux transporter HMPRNC0000_2472 were upregulated.

Transporters of inorganic solutes were significantly activated. These systems include iron and siderophore uptake genes *ceuB* and *sirA*, an operon of ABC iron uptake permeases HMPRNC0000_0811-0813, and ferrichrome binding periplasmic protein HMPRNC0000_2507. Various Na^+^-symporters, *kefC*, *mnhC*, *mnhD*, *nhaC*, and HMPRNC0000_0689, as well as K^+^-uptake proteins (*ktrA* and *ntpJ2*) and alkanesulfonate transporter *ssuC* were activated. Potassium transport system Ktr plays an important role in maintenance of the osmotic homeostasis in S. aureus (37). Other osmoprotective compounds, glycine betaine and polyamines, were actively synthesized (*betB*) or transported to the cell (*opuD2*, *potA*, *potC*). Osmotic stress may occur due to damaging of the bacterial cell walls by iodine. Genes involved in cell wall biosynthesis were regulated differentially. The peptidoglycan synthesis, salvage, and stress response genes, *N*-acetylmuramoyl-l-alanine amidase *atl*, peptidoglycan dd-endopeptidase *dacB*, cell wall stress response autolysin *amiD2*, and cell wall biosynthesis genes *fmtB2*, *murD*, and *murE* were upregulated. In contrast, d-alanylalanine ligase *ddl*, peptidoglycan transglycosylase *mgt*, *N*-acetylmuramic acid 6-phosphate etherase *murQ*, and *N*-acetylmuramic acid-specific transporter *sacX* were downregulated.

### Differential gene expression in A. baumannii ATCC BAA-1790.

Gene regulation in A. baumannii treated with FS-1 in the Lag and Log growth phases is shown in Table S3. The scheme of the regulated metabolic pathways is shown in Fig. 2C.

Exposure of A. baumannii to FS-1 has activated the transcription of citrate synthase *gltA-2* and isocitrate lyase *aceA* involved in TCA and glyoxylate cycles. These pathways were strongly inhibited by FS-1 in E. coli and S. aureus. Upregulation of phosphoenolpyruvate carboxykinase *pck* indicated an activation of the gluconeogenetic pathway, whereas the glycolysis was not activated by FS-1, in contrast to the two former model microorganisms. The activation of the anabolic gluconeogenetic pathway may be associated with the intensified synthesis of various amino acids observed in the FS-1-treated A. baumannii. Genes associated with synthesis of branched-chain amino acids (*ilvC*, *ilvH*, *ilvI*, *leuB*, *leuC*, *leuD*), aromatic amino acid (*aroF*), cysteine (*cysD*, *cysI*, *cysN*), arginine (*argG*), glutamate (*gltD*), methionine, lysine, and threonine (*metE*, *metB*, *usg*), and *S*-adenosylmethionine (*metK*, *mmuM*) were upregulated at least in one or both growth phases. These pathways were generally inhibited in the FS-1-treated E. coli and S. aureus.

Cytochrome *bd*-I terminal oxidase CydAB showing an upregulation in the FS-1-treated E. coli and S. aureus was suppressed in the FS-1-treated A. baumannii. In the text above, it was hypothesized that the activation of this cytochrome complex together with TCA downregulation and glycolysis activation might be indicative for the transition of bacteria to anaerobic respiration, which possibly was not the case with the FS-1-treated A. baumannii. Observed upregulation of the aerobic fatty acid oxidation pathway (*fadAB*) and downregulation of the anaerobic branch of this pathway (*fadK*) corroborate this hypothesis.

Genes involved in arginine catabolism to ammonia (*astA*, *astB*, *astD*, *astE*) were downregulated that opposed the influence of the FS-1 treatment on E. coli and S. aureus. The treatment of A. baumannii with FS-1 has activated ribosomal inhibitors *rsfA* and *yfiA*, while the pattern of upregulated and downregulated ribosomal proteins was dissimilar to that in the FS-1-treated E. coli and S. aureus, with many of these genes being strongly downregulated. Genes *pyrH*, *lpxH*, and *fabF* involved in the nucleotide and fatty acid biosynthesis were downregulated, while the nucleotide degradation and salvage genes *ppnP* and *rutF* were upregulated. A strong downregulation was observed for potassium uptake proteins KdpABCD, which play an important role in maintenance of osmotic homeostasis (38, 39) and in adaptation of E. coli to the presence of FS-1 in the medium (26). Peptidoglycan synthesis and assembly genes *murI* and *dacA* and heavy metal resistance genes *copA* and *spxA* were strongly upregulated in FS-1-treated A. baumannii as was observed also in the FS-1-treated E. coli and S. aureus.

The activation of many genes involved in the thioredoxin pathway, glutathione-glutaredoxin redox reactions, and oxidative stress response (*gntY*, *nfnB*, *usg*, *nemA*, *osmC*, and *wrbA-2*) suggests that the FS-1-treated culture of A. baumannii experienced an oxidative stress. In the FS-1-treated E. coli and S. aureus, the homologous genes were not regulated, while other genes controlling the redox homeostasis showed some levels of downregulation: *grxA* and *trxC* in E. coli and *gpo* and *osmC* in S. aureus.

Multiple genes involved in iron scavenging and uptake were activated in the FS-1-treated A. baumannii, while gene *bfr-1* for iron storage in bacterioferritin was downregulated. The sulfate and thiosulfate uptake and assimilation genes (*cysA*, *cysB*, *cysP*, *cysW*, and *cysU*) were strongly upregulated in the Log phase, which probably was associated with an activation of biosynthesis of sulfate-containing amino acids cysteine, methionine, and *S*-adenosylmethionine. The same was true for the observed upregulation of *tauAB* controlling uptake and assimilation of sulfate-rich compound taurine. Nitrate/sulfonate/bicarbonate ABC transporter *ybaN* collocated on the chromosome with the *tauAB* operon was activated, also. In the Lag phase, all these genes were strongly downregulated. In contrast, phosphate ABC transporter permease subunits *pstA* and *pstB* were downregulated by the treatment with FS-1 in the Log phase but upregulated in the Lag phase. Several other genes showed an opposite regulation in the Lag and Log experiments on A. baumannii including sigma factor *rpoH* and transcriptional regulator *merR*, which were upregulated by the treatment with FS-1 in the Lag phase but downregulated in the Log phase. Differential expression of these top-level transcriptional regulators may be behind the opposite regulation of other above-mentioned genes in the Lag and Log experiments.

### Transcriptional regulation of the genes located on the plasmids of E. coli ATCC BAA-196 and A. baumannii ATCC BAA-1790.

Genomes of the two model microorganisms used in this study, E. coli BAA-196 and A. baumannii BAA1790, comprise one plasmid of 266,396 bp and two plasmids of 67,023 bp and 10,955 bp, respectively. There are 264 predicted protein coding genes on the E. coli plasmid, but almost all of them were transcriptionally silent under the experimental and the negative-control conditions. Low-level transcription was recorded only for 8 transposases, two beta-lactamases and two aminoglycosidase acetyltransferases. The treatment of these bacteria with FS-1 in the Lag phase suppressed the transcription from all these genes, and they showed a differential regulation in the Log growth phase. However, the observed changes in the levels of gene expression in both experiments were statistically unreliable with *P* values much higher than 0.05. A previously published study, where the strain E. coli BAA-196 was cultivated with FS-1 for 10 passages, showed a significant expression from almost all the genes located on the plasmid, suggesting its importance for protection of this bacterium from environmental stresses (27). Possibly, the 5-min treatment with FS-1 was insufficient for the plasmid activation.

The A. baumannii plasmids comprise 82 protein coding genes on the larger plasmid and 13 genes on the shorter one. Almost all these genes showed some level of expression when the strain was cultivated on the medium without FS-1. Many of these genes were differentially regulated by FS-1. The highest level of up- and downregulation was observed for the genes encoding plasmid conjugation proteins of the larger plasmid. However, in all these cases the observed differential gene expression was statistically unreliable due to significant variations in the levels of expression of these genes in repeated experiments. Two genes of the shorter plasmid of A. baumannii BAA-1790 were regulated by FS-1 in the Lag experiment: a gene for TonB dependent receptor was 2-fold upregulated, and a conserved uncharacterized gene was 2-fold downregulated. No significant regulation was observed for the genes of this plasmid in the Log experiment.

### Comparison of gene expression profiles of three model microorganisms treated with FS-1.

To compare the patterns of gene expression, the program GET_HOMOLOGUES (38) was used to identify homologous genes shared by the model microorganisms. A summary of this search is represented by the Venn diagram in Fig. S2. There were 504 genes shared by the three genomes, and additionally 839 genes were common to E. coli and A. baumannii, 221 homologs were shared by E. coli and S. aureus, and 122 homologous genes were shared by S. aureus and A. baumannii.

10.1128/mSystems.01293-20.2 FIG S2 Venn diagram of distribution of strain-specific and homologous core genes shared by E. coli ATCC BAA-196, S. aureus ATCC BAA-39, and A. baumannii ATCC BAA-1790 as predicted by the program GET_HOMOLOGUES. Large admixed clusters of homologous phage-related integrases and transposases were considered strain-specific genes. Download FIG S2, PDF file, 0.8 MB. Copyright © 2021 Korotetskiy et al. 2021 Korotetskiy et al. https://creativecommons.org/licenses/by/4.0/ This content is distributed under the terms of the Creative Commons Attribution 4.0 International license.

**FIG 4:**
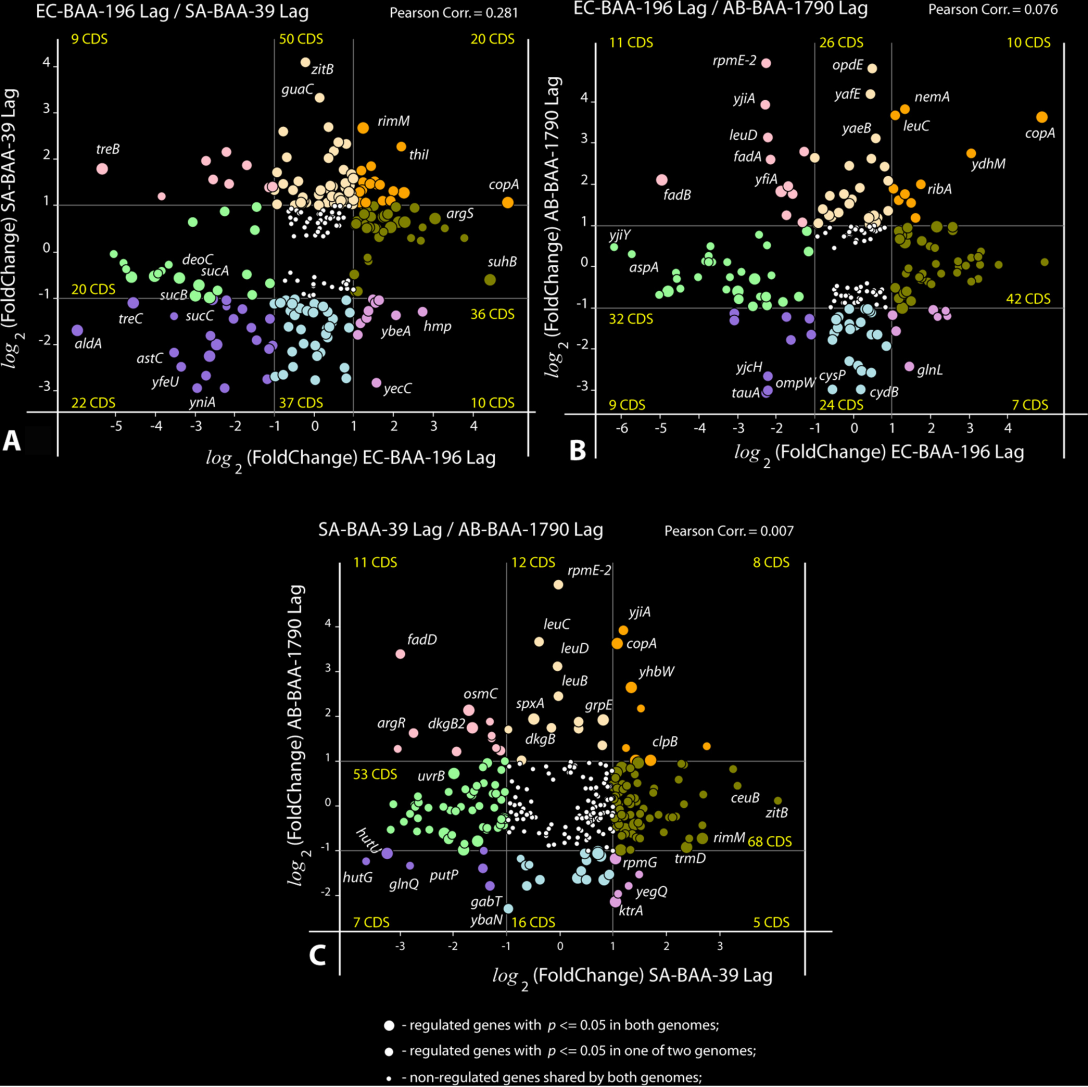
Plots of coregulation of homologous genes shared by pairs of model microorganisms in the Lag experiment. (A) E. coli BAA-196 and S. aureus BAA-39 (EC-BAA-196/SA-BAA-39); (B) E. coli BAA-196 and A. baumannii BAA-1790 (EC-BAA-196/AB-BAA-1790); (C) S. aureus BAA-39 and A. baumannii BAA-1790 (SA-BAA-39/AB-BAA-1790). Circles represent protein coding genes (CDS) plotted according to their negative and positive log_2_(fold change) values calculated in the Lag experiments for different microorganisms shown along *x* and *y* axes. The outermost regulated genes are labeled by their names. Thin vertical and horizontal lines within the plots separate genes with 2-fold or higher regulation and split the plots into sectors of genes of different categories depending on their coregulation. Numbers of CDS falling into different sectors are shown. Up- and down-coregulated genes and oppositely regulated genes are depicted by different colors. Statistical reliability of the fold change predictions is depicted by sizes of the circles as explained in the legend at the bottom of the figure. Estimated Pearson correlation coefficients are given on the top of each plot.

Plots of the gene expression fold changes determined for the homologous genes in pairs of model organisms, E. coli and S. aureus (Fig. 4A), E. coli and A. baumannii (Fig. 4B); and S. aureus and A. baumannii (Fig. 4C), all treated with FS-1 in the Lag phase, are shown in Fig. 4. Calculated Pearson correlation coefficients were in the range from 0.281 for the pair E. coli-S. aureus (Fig. 4A) to 0.007 for the pair S. aureus-A. baumannii (Fig. 4C). Only one gene was found to show a significant upregulation in all three tested microorganisms: heavy metal (copper, lead, cadmium, zinc, and mercury) transporter *copA*. It suggests that the treatment with FS-1 has increased penetrability of the cells for heavy metal ions in all three tested bacteria probably due to damage of cell wall and cytoplasmic membrane barrier molecules by iodine. In response to the influx of heavy metal ions, the corresponding efflux pumps were activated. This hypothesis was experimentally confirmed by high-pressure liquid chromatography (HPLC) measurement of intracellular antibiotic concentrations in the intact and FS-1-treated E. coli BAA-196 (27). This finding also is in agreement with another study reporting a disintegration of bacterial membranes by iodine nanoparticles (18). Other authors reported that the copper homeostasis systems may be activated in E. coli under anaerobic conditions associated with amino acid limitation (40).

The close-to-zero level of gene coregulation in these three phylogenetically distant bacteria treated with FS-1 may be explained by the fact that the regulation of similar metabolic processes often is carried out by up- and downregulation of different genes involved in the metabolic pathways. Indeed, the comparison of the affected metabolic pathways of all these bacteria (Table 3 and Fig. 2) demonstrated a higher similarity in their responses to the treatment with FS-1. This commonality includes downregulation of many transmembrane transporters of carbohydrates, amino acids, oligopeptides, and oligosaccharides in all these bacteria. FS-1 is a complex of iodine molecules bound to dextrin fragments and oligopeptides. Transporters of these compounds may serve as entrance points for iodine to the cells. This may explain the observed inhibition of these transporters; however, the spectra of inhibited and activated transporters were different in the tested bacterial strains. Sodium-proline symporter *putP* was downregulated in all three tested bacteria almost under all conditions (except for E. coli in the Log phase). Iron scavenging and uptake proteins were activated in S. aureus and A. baumannii, probably due to immobilizing of iron ions by iodine into insoluble iodides; however, in E. coli the expression of these genes was not regulated. Other systems of transmembrane uptake and utilization of various compounds were regulated differentially in the tested bacteria.

Synthesis of cell wall compounds including peptidoglycans and lipids was generally upregulated. This observation confirms that the prime target of the released iodine was the cell wall and cell membrane structures, a finding which agrees with other studies (18, 21, 27). Various efflux pumps including several heavy metal resistance proteins were activated by the treatment with FS-1. The activation of synthesis and uptake of osmoprotective molecules (glycine betaine and polyamines) and the potassium uptake systems in E. coli and S. aureus suggests that these strains experienced an osmotic stress caused by the treatment with FS-1. In E. coli, an important role in gene regulation under the experimental condition was played by the cAMP-CRP signal transduction system (Fig. 3); however, it was not the case with gene regulation in the FS-1-treated S. aureus and A. baumannii, which probably did not utilize cAMP-CRP for gene expression regulation. Particularly, adenylate cyclase *cyaA* was strongly downregulated in the FS-1-treated S. aureus.

The effect of FS-1 on A. baumannii in the Log growth phase was rather specific and characterized by a significant downregulation of many genes including several important transcriptional regulators: the EnvZ/OmrR two-component signal transduction system involved in osmoregulation (41), the RstA response regulator of many intracellular processes (42), the potassium-dependent KdpD regulator controlling K^+^-homeostasis (43), iron uptake regulator *fur*, sigma factor-32 *rpoH*, and transcriptional regulator *merR*.

## DISCUSSION

A noteworthy finding of this work was that the tested model microorganisms responded differently to the short (5-min) treatment with iodine-containing nano-micelles. The treatment of E. coli and S. aureus with FS-1 caused a transition of these bacteria to anaerobiosis. One possible reason for this reaction may consist in damage to the O_2_-dependent electron transportation systems by I_2_ molecules. This caused bacteria to look for alternative electron acceptors such as DMSO in the case of E. coli or nitrates/nitrites in the case of S. aureus. In another study on E. coli BAA-196 cultivated for 10 passages with FS-1, a complete stall of expression of genes encoding cytochrome *bo* terminal oxidase complex (*cyoA*, *cyoB*, *cyoC*, and *cyoE*) was observed (26, 28) that corroborates the hypothesis that iodine may interfere with the systems of electron transportation between cytochromes and oxygen. This transition to anaerobiosis caused by the treatment with FS-1 may be of practical importance. Both these bacteria, E. coli and S. aureus, may survive anaerobic or microaerophilic conditions; however, the transfer to anaerobiosis occurs at the expense of a reduction of their virulence and antibiotic resistance (44).

Another reason for the transition to anaerobiosis may consist in an attempt to alleviate the oxidative stress caused by iodine in the medium as the oxidative phosphorylation is a source of peroxide radicals aggravating the oxidative stress. This hypothesis is supported by the observed strong oxidative stress response in the FS-1-treated A. baumannii, which in contrast to E. coli and S. aureus did not show any signs of transition to anaerobiosis. Several other incongruent responses by A. baumannii to the treatment with FS-1 compared to the other two microorganisms, such as the activation of gluconeogenesis over glycolysis and the enzymes involved in the TCA cycle, glyoxylate pathway, fatty acid beta-oxidation, and amino acid biosynthesis with opposite effects in the FS-1-treated E. coli and S. aureus may be attributed to the transition of the latter strains to anaerobiosis with the absence of such a response in A. baumannii. The gene expression profiles in A. baumannii under the effect of FS-1 differed in the Lag and Log phases, with several genes involved in sulfur, nitrogen, and phosphorus uptake being regulated oppositely in these two experiments.

The treatment of bacteria with the iodine-containing micelle drug FS-1 caused the osmotic and oxidative stresses to the bacterial cells. The osmotic stress was associated with damage to cell wall and cytoplasmic membrane proteins leading to an increased penetrability of bacterial cells by heavy metal ions and antibiotics (27). This effect was more apparent in E. coli and S. aureus. The oxidative stress could be partly alleviated in the FS-1-treated E. coli and S. aureus due to the transition to anaerobiosis. However, the strong inhibition of multiple nutrient uptake and utilization systems in E. coli could lead this bacterium to a shortage of energy and nutrients. In S. aureus, the elevated accumulation of iodine in the cytoplasm caused stronger damage to DNA that was apparent from the observed activation of DNA repair and nucleotide salvage systems. Another study in which S. aureus was cultivated for a long time with FS-1 showed an increased rate of mutations and abnormal epigenetic modifications of the chromosomal DNA (26). This activation of the DNA repair systems in response to the treatment with FS-1 was not observed in the other two bacteria. The cell wall of A. baumannii was apparently the most resistant to iodine and protected cells from iodine penetration into the cytoplasm. However, this bacterium was under a stronger oxidative stress than the other two microorganisms.

In this work, the gene expression profiles in the treated bacteria were analyzed using the total RNA extraction followed by Ion Torrent PGM sequencing. The same approach was used in the previously published studies on the effect of FS-1 on bacterial pathogens (S. aureus BAA-39 and E. coli BAA-196) during long-term cultivation with the sublethal concentrations of the drug (26, 27). Following the same experimental procedures allowed a cross-comparison of the results of the different projects. A drawback of the Ion Torrent sequencing is that the amounts of DNA reads yielded by this technique (Table 1) may not be sufficient to identify all the regulated genes with a statistical reliability (*P* value < 0.05). The lists of the regulated genes shown in Tables S1 to S3 may not be comprehensive, giving only an overview of regulation of the highly expressed genes. Particularly, the depth of sequencing achieved in this study was not sufficient to detect changes in the expression of many transcriptional factors and some other genes characterized with naturally low levels of expression. For a detailed analysis of expression of individual genes involved in the response to the treatment with iodine-containing nano-micelles, deeper sequencing or RT-PCR will be used in future studies.

### Conclusion.

The three tested pathogenic bacteria responded differently to the treatment with the iodine-containing nano-micelles depending also on the growth phase when the drug was applied. One common mechanism was the activation of efflux pump CopA, which probably points to damage to cell wall structures in the FS-1-treated bacteria leading to an increased permeability of the cytoplasmic membrane and the osmotic stress. This information will be of importance for development of new iodine-containing nano-micelle drugs against antibiotic-resistant nosocomial infections represented in this study by the reference multidrug-resistant strains E. coli ATCC BAA-196, S. aureus ATCC BAA-39, and A. baumannii ATCC BAA-1790. Further studies will aim at testing the effects of iodine-containing compounds on a broader collection of antibiotic-resistant nosocomial infection agents isolated from clinics in several recent studies (45, 46). Although iodine has been in use by humankind as an antimicrobial disinfectant for more than 2 centuries, to our best knowledge until now there have been no published reports on the effect of iodine applied in sublethal concentrations in nano-micelle complexes on gene expression and metabolism of pathogenic bacteria.

## MATERIALS AND METHODS

### Bacterial cultures.

Multidrug-resistant strains Staphylococcus aureus subsp. *aureus* ATCC BAA-39, Escherichia coli ATCC BAA-196, and Acinetobacter baumannii ATCC BAA-1790 were obtained from the American Type Culture Collection (ATCC) and used as the model organisms in this study. Stock cultures were kept in a freezer at −80°C. Bacteria were cultivated on Mueller-Hinton (MH) liquid or solid medium (Himedia, India) without antibiotics (S. aureus and A. baumannii) and in the medium supplemented with 10 μg/ml ceftazidime (E. coli) as recommended in the ATCC product sheets. Before use in the experiments, the strains were twice passaged on MH medium.

### Determination of growth rates.

Overnight (24-h) culture growth was adjusted by dilution with sterile water to 1 × 10^7^ CFU/ml and inoculated into microarray plates with MH liquid medium. Plates were incubated at 37°C on a 60-rpm shaker for 24 h with optical density (OD at 620 nm) recording every 5 min by a Multiskan Ascent spectrophotometer (Thermo, Finland). The growth curves were estimated based on the series of OD values obtained in three repeats by the Baranyi and Roberts model implemented in the interactive DMFit application (47) as shown in Fig. S1.

### Treatment with FS-1.

Bacterial inoculants were incubated at 37°C as follows: E. coli for 1 h (the end of the lagging growth phase, termed Lag experiment) and for 6 h (the middle of the logarithmic growth phase, Log experiment), S. aureus for 2.5 h (Lag) and 9 h (Log), and A. baumannii for 1 h (Lag) and 10 h (Log). Lag and Log growth phases were estimated based on the strain-specific growth curves on MH medium (Fig. S1). Thereafter, the experimental cultures were supplemented with FS-1 to achieve the final concentration of 450 μg/ml (for S. aureus) or 500 μg/ml (for E. coli and A. baumannii) that corresponded to the half of the minimal bactericidal concentrations of FS-1 estimated for these bacteria, whereas for the negative-control samples, the same volume of physiological saline was added to the bacterial cultures. Cultures were incubated for 5 min at 37°C with shaking. Metabolic processes were stopped by adding the killing buffer (2.0 ml of 1 M Tris-HCl, pH 7.5; 0.5 ml of 1 M MgCl_2_, 1.3 g of NaN_3_; 997.5 ml of water) in the volume ratio 1:1 (48). Bacterial cells were collected for RNA extraction by centrifugation at 5,000 × *g* for 10 min.

### RNA library preparation and sequencing.

Total RNA samples were extracted from the cultures with the use of the RiboPure Bacteria kit (Ambion, Lithuania) as instructed by the developer’s guidelines. Afterward, the quantity and quality of the extracted RNA were verified with the use of the NanoDrop 2000c spectrophotometer (Thermo Scientific, USA) at optical wavelengths of 260 and 280 nm. Purification of the total RNA from 16S and 23S rRNA was carried out using the MICROBExpress bacterial mRNA purification kit (Ambion, Lithuania) following the developer’s recommendations. Thereafter, the effectiveness of sample purification was determined on the Bioanalyzer 2100 (Agilent, Germany) with the RNA 6000 Nano LabChip kit (Agilent Technologies, Lithuania).

The library preparation from the extracted RNA samples included an enzymatic fragmentation step with the use of the Ion Total RNA Seq kit V2. Thereafter, the Ion Xpress RNA-Seq Barcode 01-16 kit was used for RNA fragment barcoding. cDNA sequencing was then performed using the Ion 318 Chip kit V2 on the Ion Torrent PGM sequencer (Life Technologies, USA).

### Differential gene expression analyses.

Gene expression in bacterial cells treated with FS-1 for 5 min was compared to gene expression profiles in bacterial cells treated with the same volume of saline (negative control). RNA extraction from bacterial cells treated with FS-1 and grown under the negative-control condition was repeated three times with E. coli BAA-196 and six times with S. aureus BAA-39 and A. baumannii BAA-1790. RNA samples were collected from different test tubes with bacterial growth incubated in parallel to ensure biological rather than technical replication of the results.

Sampled RNA was converted to cDNA and sequenced as explained above. Generated DNA reads were quality trimmed and filtered using the raw DNA read processing pipeline implemented in Unipro UGENE v1.32.0 (49) using 20 as the quality threshold. Reads shorter than 30 bp were filtered out.

The differential gene expression was estimated using the R-3.4.4 software packages. First, reference indices were built for each reference genome using the *buildindex* function available in the *Rsubreads* package (Bioconductor, www.bioconductor.org). For each bacterium, the obtained RNA fragments were aligned to the relevant reference genomes in FASTA formats (S. aureus ATCC BAA-39 NC [CP033505], E. coli ATCC BAA-196 NC [CP042865 for the chromosome and CP042866 for the plasmid], A. baumannii ATCC BAA-1790 [CP042841 for the chromosome and CP042842 and CP067103 for the plasmids]). Resulting read alignment files in BAM format were sorted using the SAMtools software package available from htslib (www.htslib.org) and used for counting of reads overlapping predicted gene locations by using the *featureCounts* function and the relevant genome annotation files in GFF format. The R packages *DESeq2* (Bioconductor) and *GenomicFeatures* tools were then used for normalization of read counts and calculation of the differential gene expression statistics. All the above-mentioned tools were pipelined using an in-house Python 2.7 script available from the SeqWord project website http://seqword.bi.up.ac.za/transcriptomics_scripts/. This script also was used for visualization of the results as shown in Fig. 1 and 4.

The numbers of quality-controlled DNA reads generated in different experiments and the numbers of reads uniquely mapped against coding sequences (CDS) and rRNA sequences of the reference bacterial genomes are summarized in Table 1.

### Metabolic pathway and regulatory network analyses.

With the use of identified differentially expressed genes, metabolic pathways and reactions controlled by those genes were identified using the Pathway Tools v24.0 software (50). Networks of regulated genes were constructed using the web-based tool PheNetic (51) based on the regulation network designed for E. coli K-12 (NC_000913.2) and available from the PheNetic website. Pairs of homologous genes in the K-12 and BAA-196 genomes and also the homologous genes shared by S. aureus BAA-39, E. coli BAA-196, and A. baumannii BAA-1790 were identified using the program GET_HOMOLOGUES with the parameters set by default (38). Large admixed clusters of homologous phage-related integrases and transposases were considered strain-specific genes and excluded from the transcription comparison. A Venn diagram with numbers of shared genes is shown in Fig. S2.

### Statistical evaluations.

All RNA sequencing experiments were performed in six (S. aureus and A. baumannii) or three (E. coli) repetitions. Genes showing a 2-fold or greater expression difference with calculated *P* values equal to or smaller than 0.05 were considered significantly regulated. Aggregated Pearson correlation coefficients (*C_p_*) of gene coregulation were calculated by equation 1 implemented in an in-house Python 2.7 script mentioned above.

(1) $$C_{P}=\frac{N\sum^{}x_{i}y_{i}-\sum^{}x_{i}\sum^{}y_{i}}{\sqrt{\left(N\sum^{}x_{i}^{2}-{\left(\sum^{}x_{i}\right)}^{2}\right)\left(N\sum^{}y_{i}^{2}-{\left(\sum^{}y_{i}\right)}^{2}\right)}}$$

where *x_i_* and *y_i_* are fold change values estimated for every *i*’s homologous gene and *N* is total number of shared homologous genes.

### Data availability.

Experimental and negative-control RNA reads generated in this study are freely available from BioProject websites of the reference genomes deposited at NCBI: S. aureus BAA-39 NC, PRJNA480363; E. coli BAA-196 NC, PRJNA557356; A. baumannii BAA-1790 NC, PRJNA557366.
