# Supplementary figures and images for: Comparison of Transcriptional Responses and Metabolic Alterations in Three Multidrug-Resistant Model Microorganisms, Staphylococcus aureus ATCC BAA-39, Escherichia coli ATCC BAA-196, and Acinetobacter baumannii ATCC BAA-1790, on Exposure to Iodine-Containing Nano-micelle Drug FS-1
Source: mSystems. 2021 Mar 16;6(2):e01293-20. doi: 10.1128/mSystems.01293-20 (PMC8547003; doi:10.1128/mSystems.01293-20)

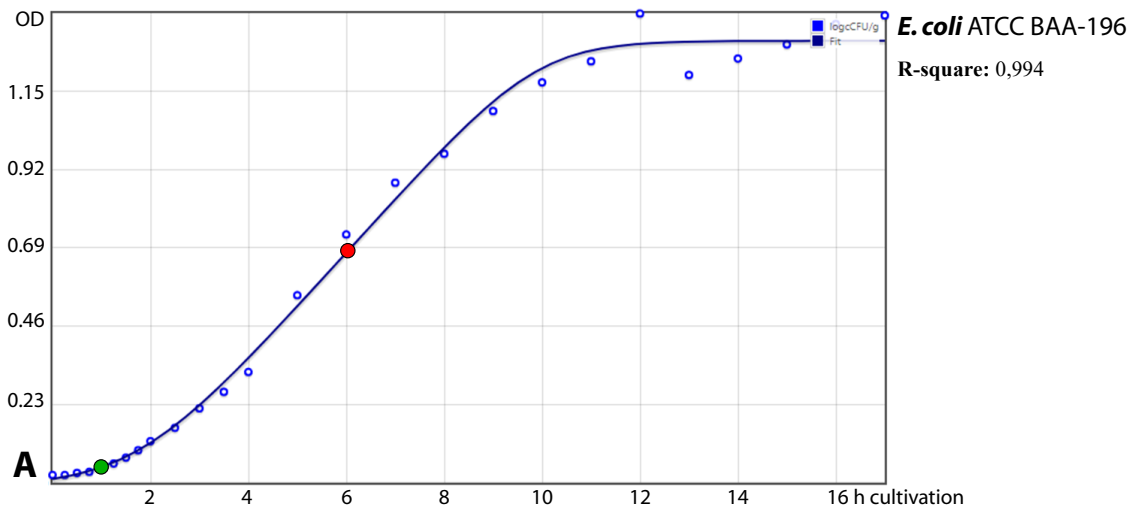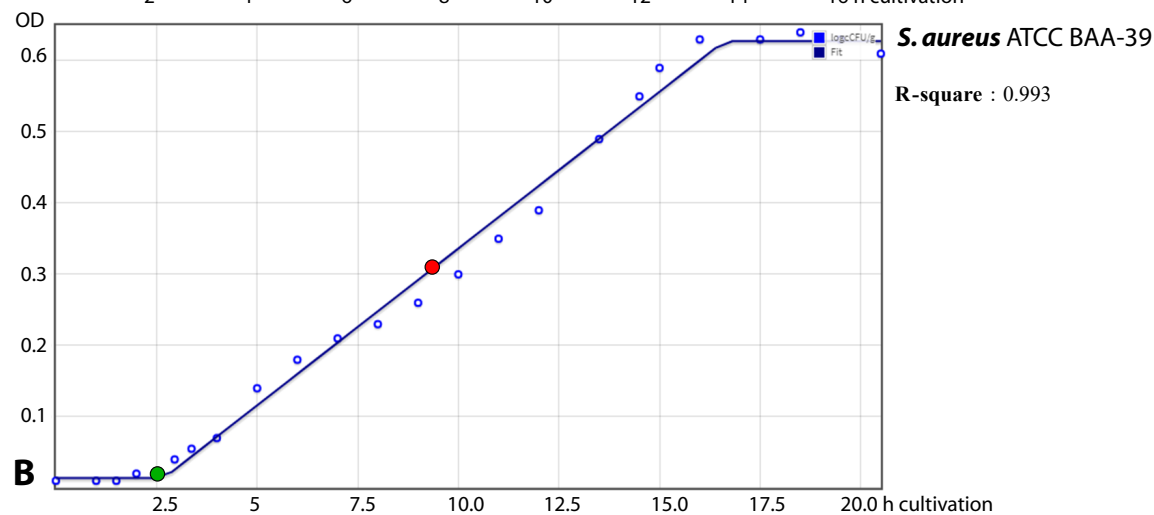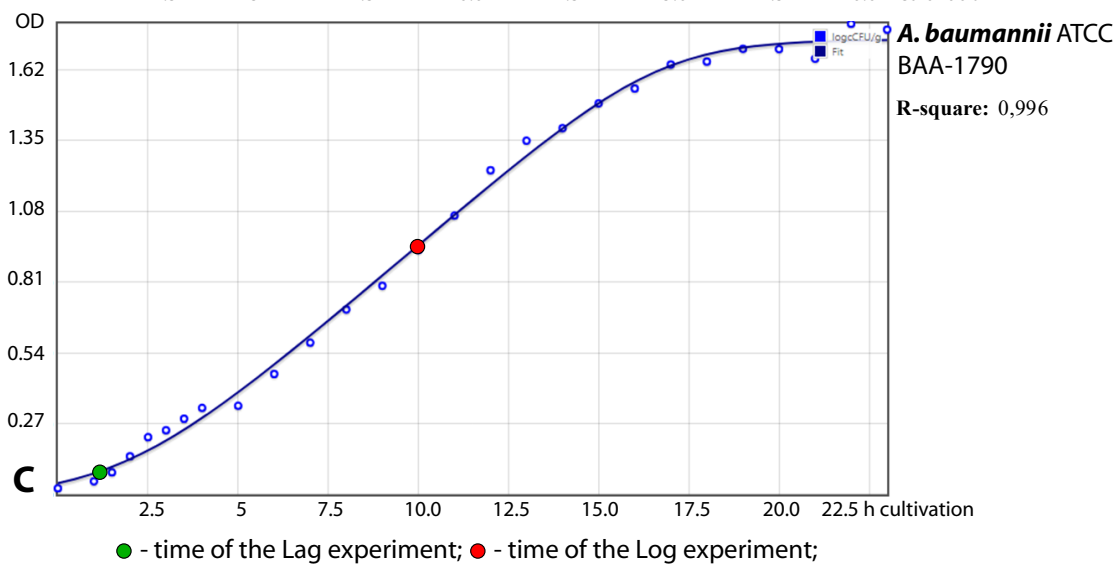

Supplement: FIG S1 [file msystems.01293-20-sf001.pdf]

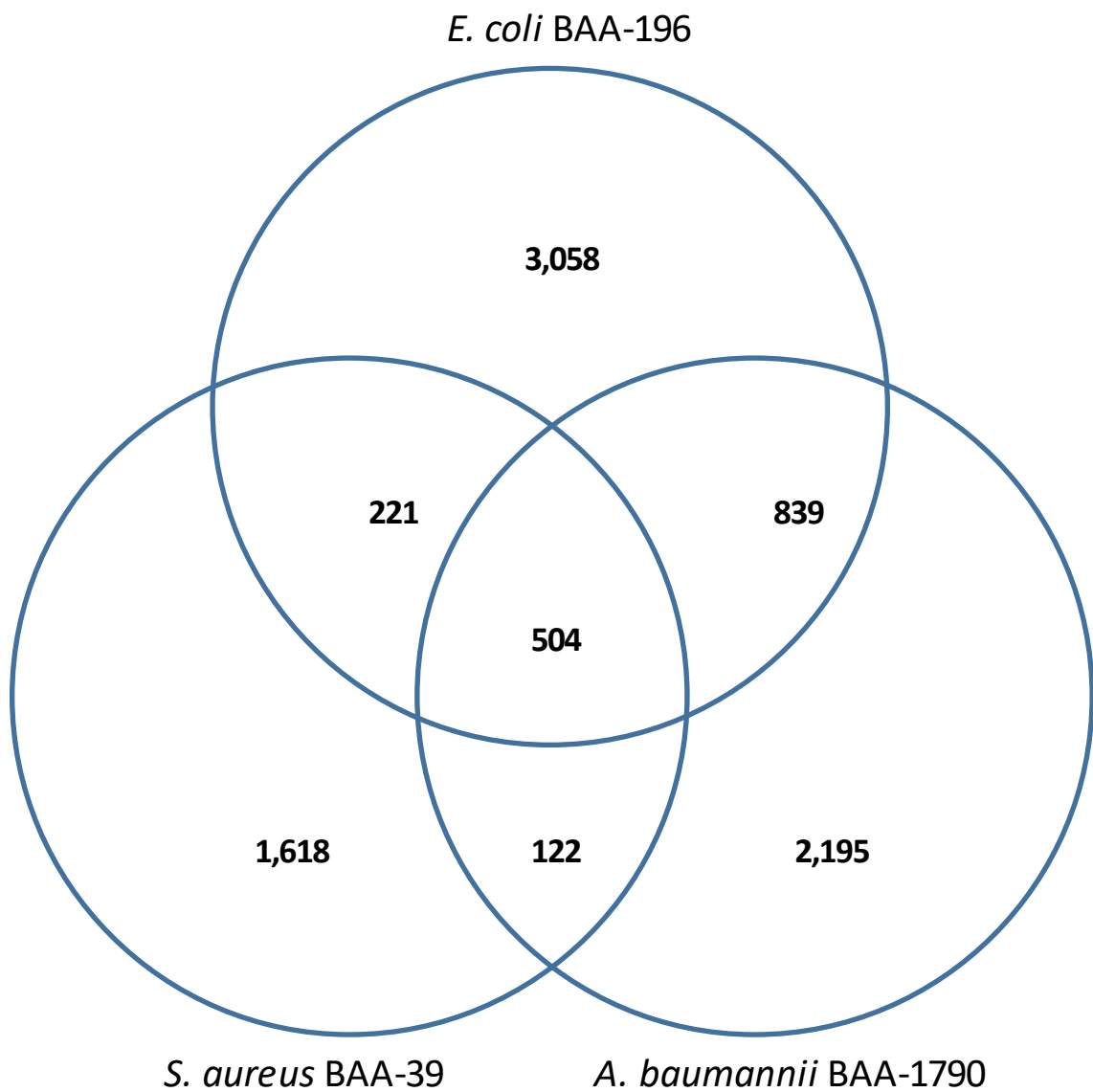

Supplement: FIG S2 [file msystems.01293-20-sf002.pdf]
